# Does evaluative scientometrics lose its main focus on scientific quality by the new orientation towards societal impact?

**DOI:** 10.1007/s11192-016-2200-2

**Published:** 2016-12-03

**Authors:** Lutz Bornmann, Robin Haunschild

**Affiliations:** 1 Division for Science and Innovation Studies, Administrative Headquarters of the Max Planck Society, Hofgartenstr. 8, 80539 Munich, Germany; 2 Max Planck Institute for Solid State Research, Heisenbergstraße 1, 70569 Stuttgart, Germany

**Keywords:** Scientific revolution, Research impact, Research quality, Bibliometrics, Altmetrics, Societal impact

## Abstract

When the meaning of key terms is incompatible in competing taxonomies, a revolution might occur in the field by which the established taxonomy is replaced with another. Since the key term “impact” in scientometrics seems to undergo a taxonomic change, a revolution might be taking place at present: Impact is no longer defined as impact on science alone (measured by citations), but on all sectors of society (e.g. economics, culture, or politics). In this Short Communication, we outline that the current revolution in scientometrics does not only imply a broadening of the impact perspective, but also the devaluation of quality considerations in evaluative contexts. Impact might no longer be seen as a proxy for quality, but in its original sense: the simple resonance in some sectors of society.

## Introduction

In recent years, one was generally interested in two measurements in evaluative scientometrics: (1) How productive was the researcher, research group, or institution in terms of the number of publications in the last few years? (2) Did these publications have high quality? The focus of evaluative scientometrics was especially directed at the quality of publications which was measured as a rule in terms of the number of citations.

In the 1980s and 1990s, researchers in the area of scientometrics have investigated whether citations are really able to measure quality (in the context of evaluative bibliometrics). For example, MacRoberts and MacRoberts (1986, 1987, 1988, 1997, 2010) conducted several studies on whether scientists in fact cite those publications which have influenced their own publications. If there had been a cognitive influence of the cited to the citing publication, this would have been a strong hint that citations really reflect quality: Only high-quality research should have cognitive influence. In their studies, MacRoberts and MacRoberts found however three citation patterns (and not just one dominating):

1. some publications were used but were either never cited or cited rarely;
2. some publications were cited mainly or only through secondary sources;
3. some publications were credited every time they were used.

Taken as a whole, their studies (and many other scientometric studies on this issue) do not support the assumption of evaluative bibliometrics that scientists only cite publications which had cognitively influenced them. Instead, many results gave support to the idea that giving credit to the cognitive influence by the research of other scientists is one motive besides other motives for citing.

Another line of research in scientometrics (besides citing behavior studies) which investigated the relationship of quality and citations has correlated peer assessments with citation counts. The authors of these studies argued that peer assessments are the best possible way of measuring quality of research (although these assessments also have their shortcomings, see Bornmann 2011). The comparison of citation counts with peer assessments has been widely acknowledged as a way of validating citation impact metrics (Bornmann and Daniel 2005; Garfield 1979; Harnad 2008; Kreiman and Maunsell 2011). For example, several publications have investigated the relationship between citation impact and UK Research Assessment Exercise (RAE, now Research Excellence Framework, REF) outcomes. They revealed considerably close relationships in many subject areas like biological science, psychology, and clinical sciences (Butler and McAllister 2011; Mahdi et al. 2008; McKay 2012; Smith and Eysenck 2002). Similar results have been reported for the Italian research assessment exercise: “The correlation strength between peer assessment and bibliometric indicators is statistically significant, although not perfect. Moreover, the strength of the association varies across disciplines, and it also depends on the discipline internal coverage of the used bibliometric database” (Franceschet and Costantini 2011, p. 284). An overview of publications which show a close relationship between peer assessments (or editorial decisions) at single journals and citation metrics can be found in Bornmann (2011).

In agreement with the heterogeneous results on the relationship between citations counts and quality (studied by cognitive influence and peer assessments), two competing theories of citing behavior have been developed in past decades which are situated within broader social theories of science. The normative theory of citing behavior is based on Robert K. Merton’s sociological theory of science (Merton 1973). This theory basically states that scientists give credit to other scientists by citing publications they use and are influenced by. Thus, citations reflect cognitive influence from cited to citing publications. The use of citations in evaluative scientometrics as a proxy for quality in science is grounded in the normative theory of citing behavior. The social constructivist view on citing behavior is based on the constructivist sociology of science (Knorr-Cetina 1981). This view doubts not only the normative theory of citing behavior but also the validity of evaluative scientometrics. According to the social constructivist view on citing behavior the cognitive content of publications has scarce influence on how scientists perceive them. Scientific knowledge is socially constructed by political, financial, cultural, and other contexts of scientific activities. Thus, citations are only loosely (or not) connected to the intellectual content of publications.

The relationship between citations and quality was the central question in studying citing behavior, correlating assessments by peers and citations, as well as developing theories of citation. The great interest of scientometricians over decades in this relationship points out that scientometricians were mostly interested in impact issues in these years, because citations are probably related to quality. In this Short Communication, we outline that current changes in impact measurements (impact is no longer defined as impact on science alone, but also on all sectors of society), does not only imply a broadening of the impact perspective, but also the devaluation of quality considerations in evaluative contexts. Impact of research might no longer be seen as a proxy for its quality, but in its original sense: the simple resonance in some sectors of society.

## Scientific revolution in scientometrics

Over the last few decades, a widely accepted taxonomy has been developed in scientometrics for the use of citation data (in evaluative contexts). Here, taxonomy is defined as a roughly outlined scheme which is used by scientometricians for the purposes of their research and its application (in evaluations) (Wray 2011). In the recent overview of Waltman (2016) on citation impact indicators several definitions of central terms can be found (e.g. size-dependent and size-independent indicators or percentile-based indicators). The taxonomy is used by the proponents as well as the opponents of citation analyses. The last few decades in scientometrics can be described as Kuhn (1962)’s normal science in a mature field which has seen refinements in techniques (e.g. refinements of field-normalized indicators), reworking (e.g. recurring studies on citation impact comparisons of countries) and enhancements (e.g. the development of complex variants of the well-known h index, see Bornmann et al. 2011), but no fundamental taxonomy changes.

Scientific revolutions are characterized by taxonomic changes in a mature research field (Kuhn 1962; Wray 2011). When the meaning of key terms is incompatible in competing taxonomies, a revolution might occur in the field in which the established taxonomy is replaced with another. Since the key term “impact” in scientometrics seems to undergo a taxonomic change, a revolution might be taking place at present (Bornmann 2014, 2016). Impact might no longer be seen as a proxy for quality, but is defined and used in a broader meaning which is more focused on influence or resonance. In other words, the measurement of quality aspects of research might no longer be the focus of evaluative scientometrics. The taxonomic change started to take place, when people began to understand impact increasingly in a broad way, as societal impact: Broad impact implies not only impact on science but also impact on other sectors of society. Kuhn (1962) calls such changes in the meaning of key terms ‘meaning-incommensurabilities’ between two different taxonomies (Wray 2011).

Scientific impact measurement is a part of broad impact measurement. Scientometricians know rather well what quality of research regarding science means, but the meaning of quality of research regarding other parts of society is not well known, although broad definitions for societal impact have been provided. For example, Wilsdon et al. (2015) define societal impact of research as follows: “Research has a societal impact when auditable or recorded influence is achieved upon non-academic organisation(s) or actor(s) in a sector outside the university sector itself—for instance, by being used by one or more business corporations, government bodies, civil society organisations, media or specialist/professional media organisations or in public debate. As is the case with academic impacts, societal impacts need to be demonstrated rather than assumed. Evidence of external impacts can take the form of references to, citations of or discussion of a person, their work or research results” (p. 6). Whereas the recipients of publications in science are experts who can assess the quality of cited publications, recipients outside the science area are as a rule not able to do this. Thus, the focus on the quality of publications which was an integral part of impact measurements might erode.

**Fig. 1:**
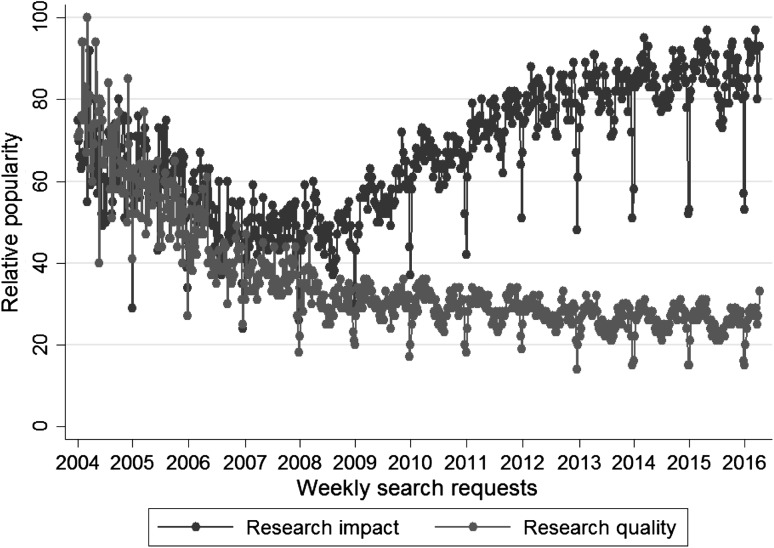
Weekly search requests on Google for the terms “Research impact” and “Research quality” between the beginning of 2004 and 2016

The results in Fig. 1 might confirm this erosion. The figure shows weekly search requests on Google for the terms “Research impact” and “Research quality” between the beginning of 2004 and 2016 (earlier data is not available). This kind of data is offered by Google in the application Google Trends. The visualized percentages show the weekly number of search requests relative to the total searches: “Google Trends adjusts search data to make comparisons between terms easier. Otherwise, places with the most search volume would always be ranked highest. To do this, each data point is divided by the total searches of the geography and time range it represents, to compare relative popularity. The resulting numbers are then scaled to a range of 0–100” (https://support.google.com/trends/answer/4365533?hl=en). It is clearly visible that until 2007/2008 the interest in “Research impact” and “Research quality” was similar. However, in recent years research impact seems to draw much more interest than research quality.

## Alternative metrics and societal impact

The taxonomic change (from quality to impact) has consequences for the work of scientometricians. New data sources for measuring societal impact come into play (Fausto et al. 2012). For example, alternative metrics (altmetrics) have been proposed for measuring societal impact, which are offered by new companies, such as Altmetric, Plum Analytics, or ImpactStory. “Altmetrics are usually based on activity on social media platforms, which relates to scholars or scholarly content. Typical examples of altmetrics include tweets, mentions in blog posts, readership counts on Mendeley, posts, likes and shares on social networks such as Facebook and Google Plus, and recommendations and ratings on F1000. However, altmetrics also comprise mentions in mainstream media or policy documents, as well as usage metrics such as full text views and downloads, although these have been available long before the concept of altmetrics was introduced. The common denominator of these heterogeneous metrics is that they exclude, and are opposed to, ‘traditional’ bibliometric indicators” (Work et al. 2015).

Altmetrics are still at a very early stage of development and scientometrics have started to explore their meaning and possible use in an evaluative framework. However, it seems to be clear that altmetrics like tweets and readership counts (on Mendeley) might reflect impact of publications in society, but are only loosely or not connected to quality of research (Bornmann 2015). Wilsdon et al. (2015) report on the result of a survey where 19 respondents proposed that altmetrics could already be used as a tool for research assessment. However, 12 respondents argued that altmetrics cannot be used since its connection to research quality is not clear. Taylor (2013) see the current period in scientometrics “far from a revolutionary step in how we measure, appraise, and understand scholarly impact in society” (p. 27). He predicts that it will take 20 years until it will be possible to measure reliably and validly of how research influences society as a whole.

## Discussion

In this Short Communication, we have outlined that the current revolution in scientometrics does not only imply a broadening of the impact perspective, but also the devaluation of quality considerations in evaluative contexts. Impact might no longer be seen as a proxy for quality, but in its original sense: the simple resonance in some sectors of society. This is an alarming development, because fraudulent research is definitely of low quality, but is expected to have great resonance if measured in terms of altmetrics. For example, Twitter is a well-known resonance medium for sensational events. Real-time visualizations of twitter messages (twitter stream tools) can show how negative events produce a great resonance in the community.

In the development of advanced indicators for broad impact measurements, the quality of research should be taken into consideration. Only those indicators should be fostered and developed further on, which can also reflect the quality of research. In the context of societal impact measurements, quality should not be evaluated in an academic sense but in the context of its possible societal benefits. In other words, research should be accurately done, but does not have to be excellent in order to be useful in a certain sector of society. Indicators which might not only reflect societal impact, but also research quality (in a specific context) are citations of scholarly publications in clinical guidelines (Thelwall and Maflahi 2015), patents (Kousha and Thelwall in press), and policy documents (Bornmann et al. 2016). Here, we can assume that the recipients of research can reliably assess the relevance of publications in a practical context.

Subsequent to a revolutionary phase, in which the new taxonomy gains acceptance in a certain community, a phase of normal science follows in which corresponding methods and techniques are newly developed or established ones adapted. In scientometrics, the phase of normal science will face the experts with questions around the methods for the reliable and valid measurement of societal impact. The specific contribution of altmetrics to the measurement of societal impact will be clarified.
